# The Genetic Complexity of Prostate Cancer

**DOI:** 10.3390/genes11121396

**Published:** 2020-11-25

**Authors:** Eva Compérat, Gabriel Wasinger, André Oszwald, Renate Kain, Geraldine Cancel-Tassin, Olivier Cussenot

**Affiliations:** 1 CeRePP/GRC5 Predictive Onco-Urology, Sorbonne University, 75020 Paris, France; g.cancel@cerepp.org (G.C.-T.); olivier.cussenot@aphp.fr (O.C.); 2 Department of Pathology, Hôpital Tenon, Sorbonne University, 75020 Paris, France; 3 Department of Pathology, Medical University of Vienna, 1090 Vienna, Austria; gabriel.wasinger@meduniwien.ac.at (G.W.); andre.oszwald@meduniwien.ac.at (A.O.); renate.kain@meduniwien.ac.at (R.K.); 4 Department of Urology, Hôpital Tenon, Sorbonne University, 75020 Paris, France

**Keywords:** prostate cancer, germline mutations, somatic mutations, PTEN, TMPRSS2, ERG, androgen receptors

## Abstract

Prostate cancer (PCa) is a major concern in public health, with many genetically distinct subsets. Genomic alterations in PCa are extraordinarily complex, and both germline and somatic mutations are of great importance in the development of this tumor. The aim of this review is to provide an overview of genetic changes that can occur in the development of PCa and their role in potential therapeutic approaches. Various pathways and mechanisms proposed to play major roles in PCa are described in detail to provide an overview of current knowledge.

## 1. Introduction

Prostate cancer (PCa) is a major concern in public health, with more than 1.1 million cases worldwide detected every year [1]. Several risk factors for developing PCa are known, e.g., older age, family history and African ethnicity. Despite the refinement of existing treatments and emergence of new management strategies, such as active surveillance and focal therapy, metastatic disease is frequent and mortality is still relatively high, with 26,730 estimated deaths in 2017 [2].

Based on the severity of disease at diagnosis according to differentiation, extension and stage, PCa may be treated in different ways; of particular importance is its initial hormone dependency which allows specific treatments, especially during early-stage disease [1]. Further differences exist between primary, metastatic (mPCa) and castration resistant PCa (CRPCa). Therefore, it is important to know the most important actors, especially the important role of genetics in this hormone-sensitive cancer.

Several studies showed many different genetically distinct subsets of PCa. Various drivers are known, such as androgen-related fusions of ETS-related gene (ERG) and ETS family members, speckle-type pox virus and zinc finger protein (SPOP) mutations, DNA hypermethylation, PIK3/RAS/RAF pathway alterations and DNA damage repair (DDR) pathways.

For better understanding, it is necessary to mention genetic screening, which was explored in the early 2000s and abandoned in 2012 after it was demonstrated that many of the detected PCa were clinically insignificant and did not affect patient life expectancy [3]. In recent years, powerful genetic tests were developed that provide polygenic risk scores for individual patients [4,5]. However, a remaining challenge is the recrudescence of clinically relevant PCa, which may also benefit from personalized approaches for risk assessment or therapy.

Despite recent advances, standard pathology remains a fundamental tool in managing PCa. The Gleason score (GS), reflecting tumor differentiation, is a staple of clinical decision-making, and recent meetings could refine the consensus and diminish interobserver variability [6]. Nevertheless, GS alone will not give all the necessary information; molecular profiling of PCa could provide further information. For instance, a study by Haffner et al. [7] showed that metastasis was not strictly and may result from a tumor region with lower grade, alongside observing PTEN loss. Clinicians need to be increasingly aware that although classical histopathology is a firmly established basis for clinical decisions, it is not the single determinant of PCa behavior.

In recent studies taking a scrutinous approach to Gleason grading of PCa, pathologists showed that cribriform and intraductal PCa, which should be considered Gleason grade 4, were probably more aggressive than the classical Gleason grade 4 pattern. Moreover, several studies underlined a more aggressive behavior of cribriform PCa, partly explained by the underlying molecular aberrations in these tumor patterns. A recent study tested genomic instability by determining the portion of the genome altered and somatic copy number alterations (CNA). Patients with cribriform and/or intraductal PCa and ≥ GS 7 had significantly higher percentages of the genome altered than men without this pattern in both cohorts of The Cancer Genome Atlas (TCGA) (2.2 fold; *p* = 0.0003) and the Canadian Prostate Cancer Genome Network (CPC-GENE) (1.7 fold; *p* = 0.004) [8]. These patterns were associated with deletions of different chromosomes, such as 8p, 16q, 10q23, 13q22, 17p13 and 1q22, and amplification of 8q24, which plays a major role in PCa evolution and is specifically addressed in this review. CNAs comprised a total of 1299 gene deletions and 369 amplifications in the TCGA dataset. Several of the affected genes were known to be associated with aggressive prostate cancer, such as loss of PTEN, CDH1 and BCAR1 and gain of MYC. Point mutations in TP53, SPOP and FOXA1 were also associated with these PCa patterns but occurred less frequently than CNAs. This study clearly shows that cribriform/intraductal patterns are associated with increased genomic instability, clustering to genetic regions involved in aggressive PCa.

**Figure 1:**
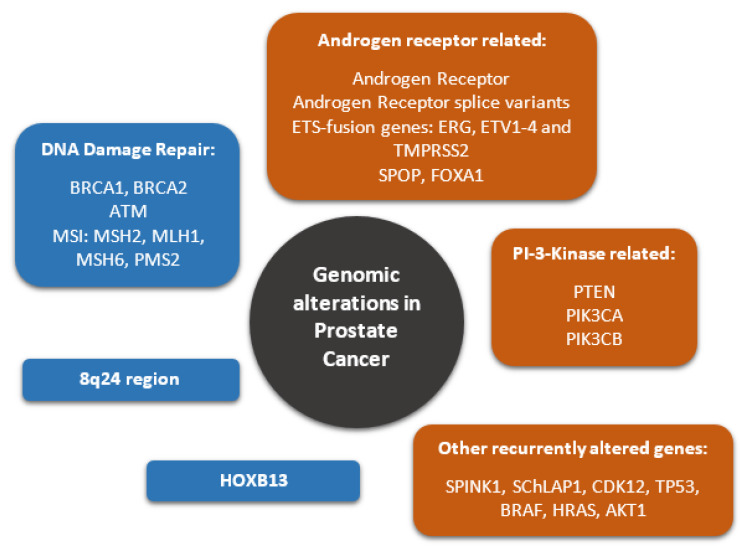
Common genomic alterations in prostate cancer. Germline mutations are highlighted in blue, while typical somatic mutations are highlighted in orange. For abbreviations see full text.

The very complex genomic situation of PCa can be broken down into two major aspects, which need to be considered, namely, the germline genetic background and the somatic changes in PCa (Figure 1).

## 2. Germline Mutations Driving PCa

Hereditary prostate cancer (HPC) is defined by strict clinical criteria and represents 5% of all newly diagnosed PCa [9]. Inherited predisposition to acquire PCa is genetically determined by the presence of a deleterious mutation of DNA repair genes also related to breast/ovarian cancers (i.e., BRCA1 and BRCA2, ATM, etc.) or PCa-specific risk genes (HOXB13 and 8q24 region) [10]. A recent study performed germline sequencing and analysis of DNA repair genes [11] in 5545 men of European ancestry, including 2775 nonaggressive (localized disease, stage T1/T2 and GS ≥ 6 tumors) and 2770 aggressive (lethal or metastatic disease, stage T4 or both T3 and GS ≥ 8) PCa cases. The authors found that BRCA2 and PALB2 showed the most statistically significant gene-based disease associations, with 2.5% of aggressive and 0.8% of nonaggressive cases carrying deleterious BRCA2 alleles, and 0.65% of aggressive and 0.11% of nonaggressive cases carrying deleterious PALB2 alleles. ATM had a nominal association, with 1.6% of aggressive and 0.8% of nonaggressive cases carrying ATM alleles. According to typology of genetic risk, predisposition exposes the individual to an earlier age of onset or a more aggressive form of the disease, increasing the risk of death from this cancer. Germline mutations of DNA repair factors are found in only up to 8% of these patients due to the rarity of the mutations [12,13].

DNA damage response (DDR) pathways are extremely promising targets in PCa treatment. Two prominent factors are breast cancer 1 and 2 genes (BRCA1 and BRCA2). The TCGA research network, testing 333 primary PCas, reported mutations in DNA repair genes in 19% of primary PCas. Among these, 3% were affected by BRCA2, including germline as well as somatic truncating mutations. Only one case displayed BRCA1 as a frameshift mutation [14]. If these tumor suppressor genes are mutated heterozygously in the germ line, they can generate aggressive forms, especially mPCa [15]. In a normal setting, BRCA1 and BRCA2 repair double-strand breaks by homologous recombination. In case of a BRCA germline mutation, a somatic loss of function in the wild-type BRCA allele is consequently frequently observed together with defective homologous recombination. Although deleterious BRCA germline variants are rare, these patients were shown to more frequently develop PCa and mPCa, and also exhibit high Gleason scores (grade group 3–5) and worse outcomes [16]. Nevertheless, these tumors did not seem to have specific histological aspects allowing them to be recognized on a standard slide. Interestingly, it was observed that ATM and BRCA1/2 germline mutations were associated with Gleason grade reclassification during active surveillance of carrier patients, with an upgrading of GS from 6(3 + 3) to GS 7(3 + 4) or 7(4 + 3) [17].

Another gene implicated in DNA repair mechanisms is ATM, which also plays a role in DNA damage repair and mediates downstream checkpoint signaling. Its prevalence in mPCa is 1.6% and therefore it must be considered, especially in these forms of PCa [15]. ATM also integrates the concept of the so-called homologous repair deficiency profile [18]. These mutations were reported in nearly 2.5% of PCa patients, some detected in lethal PCa (death due to metastatic PCa), with fewer in localized PCa (low-risk disease, GS ≤ 6, organ confined) [19]. Interestingly, in Chinese patients, ATM mutations were found in 4.5% of lethal PCa. In the mentioned American–African–Asian study, no co-mutations BRCA1/2–ATM were detected, indicating that these mutations are probably simultaneously exclusive. This study also demonstrated that mutation carriers displayed higher overall lethality, higher mPCa rates and lower PCa-specific survival in patients with diagnosed mPCa, especially in young patients under 60 years. On the other hand, the lowest carrier rate was among patients who died of PCa > 75 years or > 10 years after their initial PCa diagnosis.

The potential of detecting patients with the above-mentioned germline mutations opens the door to specific treatment approaches, one of the most promising drugs being inhibitors of anti-poly ADP-ribose polymerase (PARP). PARP inhibitors induce cell death because they interfere with a cellular mechanism of single-strand DNA break repair. These occur normally during the cell cycle, but also during oncogenesis. In case of mutation of other abovementioned repair genes, PARP is required to repair both strands, which means that the cell is entirely dependent on PARP for single-strand repair. In context of BRCA mutation and concomitant PARP inhibition, for example, a tumor cell would not be able to perform these measures, resulting in chromosomal instability, cell-cycle arrest and apoptosis [20]. A recent study by Hussain et al. [21] showed that patients with PCa harboring at least one mutation in BRCA1, BRCA2 or ATM who received the PARP inhibitor olaparib had significantly longer survival than those who received enzalutamide or abiraterone plus prednisone as control therapy. Other studies also demonstrated that metastatic, castration-resistant PCa (mCRPCa) with BRCA2 germline mutations or deleterious variants showed greater response to platinum-based chemotherapy [22]. This relationship between genotype and responsiveness to platinum chemotherapy was also observed among BRCA2 patients with breast and ovarian cancers. Another study showed concordant results, underlining that biallelic BRCA2 inactivation in mCRPCa could serve as a biomarker for predicting sensitivity to platinum chemotherapy, which showed a clear benefit in biallelic, BRCA-mutated patients [23].

These types of studies underline the necessity of germline testing in special patient groups. Considerations exist regarding who to test for genetic counseling, such as a known mutation in a cancer-susceptibility gene within the family, mPCa, high-risk, localized PCa (grade group 4/5, PSA ≥ 20, WHO group ≥ 3), young age at PCa detection, family history suggestive of Lynch syndrome and hereditary breast ovarian or prostate cancer. Prostate cancers with deficiency of the DNA mismatch repair (MMR) system are rare, with 1% at the localized stage and 5% in the advanced stages [24]. PCa rarely occurs as an unconventional malignancy of the Lynch syndrome spectrum (HNPCC) [25]. These forms of PCa have aggressive anatomopathological criteria, with intraductal forms (25%), a high incidence of Gleason score 8 and high rates (40–50%) of metastatic de novo disease which are frequently visceral (30% of metastatic patients). At the molecular level, they are characterized by a high rate of mutation (tumor mutational burden). Considering their advanced stage, they respond relatively well to androgenic deprivation, but less so to taxanes once castration-resistant. Afflicted patients are a subgroup currently considered candidates and evaluated for anti-PD1 or anti-CTL4 immunotherapy [26,27]. On the germinal level, Pritchard et al. [15] reported that, of 692 patients with mPCa, 12% showed deleterious germline mutations (1% involving MSH2, 1% MSH6 and 2% PMS2). Abida et al. [26] reported that, of 1346 PCa patients, 3% showed high microsatellite instability, of which 22% possessed a germ mutation in a gene associated with Lynch syndrome. Most patients (46%) had MSH6 mutations.

## 3. 8q24 Region

8q24 is a hotspot of susceptibility loci for PCa. These risk loci, identified by genome-wide association studies (GWAS), do not affect the coding DNA and are frequently associated with single-nucleotide polymorphisms (SNPs). It was shown that amplification of 8q24 (harboring MYC) is frequent. Often, enhancers such as rs-6983267 interact with MYC and alter sensitivity to certain crucial signaling pathways, e.g., WNT [28,29]. With ongoing research, it is evident that 8q24 variants play a role in PCa carcinogenesis. A recent meta-analysis determined significant associations between PCa risk and 15 variants in 8q24. The 8q24 region is dense with SNPs; some of these variants might enhance genes implicated in PCa carcinogenesis [29].

Although the inherited component for PCa was previously acknowledged, the identification of genetic variation on 8q24 conferring cancer-specific susceptibility may help to improve screening strategies. Genome-wide studies reported low penetrance of signals influencing the risk. Nevertheless, since risk alleles are relatively common in the population, their cumulative impact is potentially substantial. One recent study underlined several independent signals in different regions and yielded a biological annotation enriched with different elements, such as promoters, enhancers and transcription factor-binding sites, such as AR, ERG and FOXA1 [30]. In another study by the same group, 12 independent risk signals with different variants were described, some of them for the first time (rs1914295, rs190257175 and rs12549761). They were weakly correlated with already known PCa risk markers. On the other hand, this study showed that men with a cumulative risk score had a greater risk of developing PCa than the average population. The described 12 variants accounted for around 25% of what could be explained as familial genetic risk factor, highlighting the contribution of germline variation on 8q24. However, although the 8q24 region is now established as a major region for PCa susceptibility, the underlying biological mechanisms still require further elucidation [10].

## 4. Somatic Mutations Driving PCa

Outlier androgen-regulated genes play an important role in PCa development. These genes are generally expressed at low levels, but can show variation in expression of different genomic subsets [31,32]. Especially in light of emerging personalized medicine, it is increasingly important to take into consideration the individual mechanisms driving aggressive PCa.

The gene fusions in PCa are mostly controlled by androgen, fusing to members of the ETS (E26 transformation-specific) family of transcription factors [33]. One of the most cited gene fusions in PCa is the overexpression of TMPRSS2–ERG (T2E) gene fusion. A consequence of this fusion is an overexpression of oncogenic factors; this is frequently present globally, in about 50% of PCa [33]. TMPRSS2 also fuses to other ETS family genes, such as ETV1, ETV4 and, rarely, ETV5. From a morphological point of view, these tumors frequently display particular aspects, such as macronuclei, signet cell rings, cribriform aspects and intraductal carcinoma. The prognostic impact of T2E is still a matter of controversy. T2E acts as an aberrant transcription factor with oncogenic properties. Several papers indicated that its presence was an indicator of aggressive phenotype and poor prognosis [34,35,36]. Some authors claimed that it was the most important prognostic factor in patients treated with prostatectomy [37]. Furthermore, it was proposed to be a risk marker for lymph-node metastasis and poorly differentiated disease, as well as biochemical recurrence at five years [37,38]. Nevertheless, the data are still controversial, and T2E is not taken into consideration for decision-making at the moment. Several contrasting studies found no clinical significance of the TMPRSS2-ERG fusion [38,39,40]. Interestingly, recent studies showed that T2E-positive and -negative PCa are two different molecular groups of PCa, suggesting that the T2E status determines the nature of metastasis-related gene signatures [40,41]. In T2E-positive cases overexpression in mPCa was seen for several genes (GMNN, TROAP and WEE1). In T2E-negative cases, completely different metastasis-associated genes were expressed, such as ASPN, BGN and TYMS. Therefore, the authors concluded that, according to the T2E status, different genes are linked to the development of mPCa. In patients with overexpression of ASPN, BGN and TYMS, shorter event-free survivals were exhibited. TYMS (thymidylate synthetase), for instance, plays a role in DNA replication and repair. Interestingly, neither PTEN nor TP53 mutations biased the results. Finally, the study showed that the T2E status is not a strong prognostic biomarker per se, but determines the prognostic value of other biomarkers [41].

Another interesting outlier gene is SPINK1, which seems to be, in most cases, mutually exclusive with ERG overexpression [41]. SPINK1, located on 5q32, encodes a protein which functions as a trypsin inhibitor. SPINK1 positivity was shown to be an independent predictor of shorter biochemical recurrence and progression-free survival. Recombinant SPINK1 protein is able to stimulate cell proliferation in benign prostate tissue. A recent study showed that SPINK1 overexpression is linked to higher PTEN expression and lower AR (androgen receptor) expression, with the authors further suggesting that SPINK1 protein expression may not be a predictor of recurrence or lethal PCa amongst men treated by radical prostatectomy. SPINK1 and ERG proteins were not entirely mutually exclusive in this study, as some previous studies suggested [42].

Another important outlier to mention is SChLAP1, a noncoding RNA gene, located on 2q31. Apparently, SChLAP1 has no coding potential, is located in the nucleus and is associated with ETS gene fusion as well as with mPCa. SChLAP1 seems to coordinate cell proliferation and metastatic spread. High expression is associated with lethal PCa, independently of tumor differentiation (Gleason score), tumor stage, PTEN status and age. In a multivariate analysis, SChLAP1 predicted mPCa within 10 years (odds ratio = 2.45) [43].

Obviously, single gene outliers alone are limited in their results and application. These genes might become the basis of patient risk stratification and adaptations in treatment, e.g., more intense earlier treatment or targeted therapies, and will probably become more available in the upcoming years to facilitate genetic and individual subclassification [44].

## 5. Recurrently Altered Genes

PCa has a lower mutational burden than many other epithelial tumors. The recent TCGA paper showed several significantly mutated genes, such as SPOP, TP53, FOXA1, PTEN and others [14]. Clinically relevant genes, such as BRAF, HRAS and AKT1, as well as the β-catenin pathway and the DNA repair pathway (see above), also showed importance.

Loss of PTEN (located on 10q23) is frequently observed and is closely related to MYC overexpression. The latter is found on 8q24. Both together seem to play a role in high-risk PCa.

PTEN loss is associated with adverse findings in early PCa and occurs in approximatively 15% as homozygous deletions. PTEN loss or mRNA-based genomic signatures can be useful to help determine whether definitive therapy is required, and its loss seems to be more frequent in patients with African ancestry [45]. Early investigations already showed that loss of PTEN, even when detected by immunohistochemistry, was a predictor of aggressive metastatic disease. A paper by Haffner et al. [7] demonstrated that PTEN mutation was not present in morphological, higher grade lesions surrounding a tumor with PTEN loss when the Gleason score was lower. Associated TP53 and SPOP mutations were reported in the same patient. Interestingly, lymph node metastasis did not harbor the same mutations, suggesting an independent clonal/subclonal origin of these lesions. Genetically, there is strong evidence to suggest that poorly differentiated PCa (Gleason score 9/10) has a higher level of genomic instability with an increased rate of copy-number alterations and alterations in key signaling pathways (TP53, PTEN and RB1) associated with resistance to androgen deprivation therapy (ADT) [14]. The function of PTEN is closely linked to the PIK3 pathway, in which PTEN is generally considered a negative regulator. PIK3CA was shown to be frequently mutated, either via activation of mutational hotspots, coincidently activating mutations or amplification. According to the literature, PTEN-deleted tumors are likely to be PIK3CB-dependent; a coexistent loss and mutation of PTEN and PIK3CB might increase PIK3 pathway output and indicate PCa with AR signaling inhibition. PIK3 pathway and DNA repair alterations seem to be more frequent in metastatic specimens [14].

MYC overexpression is an early event in prostate cancer development. Apparently, concomitant with PTEN loss via characterized HOXB13 transcription control, genomic instability and aggressive disease with a high risk of metastases are initiated. These tumors typically show a high Gleason score and disease progression. On the other hand, isolated MYC activation and PTEN were reported to be insufficient to induce invasive PCa, with cells remaining in a precancerous stage (high-grade prostate intraepithelial neoplasia (PIN)) [46]. A recent study from Liu et al. [47] showed that gain of MYC and loss of PTEN resulted in elevated PCa mortality associated even with single copy-number changes. Regarding the interplay of these two factors, MYC overexpression can induce genetic instability, while PTEN might repress this process in PCa cells. Recent results suggested that PTEN might play a role in DNA repair, and if PTEN is lost, high levels of DNA damage that normally repress apoptosis due to increased PIK3 signaling are introduced [48].

One of the genes recurrently mutated in PCa is SPOP, an E3 ubiquitin ligase adaptor protein of the ubiquitin–proteasome system. Mutations may affect the degradation of developmental regulators of PCa, including AR. SPOP plays a role in several further important cellular functions, and intervenes in transcription, cell-cycle regulation and apoptosis.

SPOP mutations were previously described in precancerous lesions and primary tumors of the prostate, suggesting that SPOP mutations are early and recurrent events in the development of PCa [7]. SPOP mutations were identified in 6–13% of primary prostate adenocarcinomas and 14.5% of metastatic prostate cancers, but data regarding expression in distant metastasis are sparse [49]. Recent studies showed that SPOP mutations are less frequent in metastatic than in primary tumors (8% versus 11%) [14]. In the abovementioned article by Haffner et al. [7] describing the clonal evolution of a PCa in metastasis, SPOP was shown to be mutated in the lethal metastatic cell clone. In this context, it is intriguing that SPOP is also a component of the DNA damage response (DDR).

One PCa-relevant direct substrate of SPOP is the androgen receptor (AR) [50], which harbors a SPOP-binding motif. When binding to SPOP, AR undergoes ubiquitin-mediated degradation. AR splice variants that lack the SPOP-binding motif escape this degradation. Interestingly, PCa-associated SPOP mutants do not bind to AR or promote its degradation. SPOP-mediated degradation of AR is driven by antiandrogens and blocked by androgens. It is not clear whether SPOP can interact with other nuclear receptors [51].

It is unclear whether the same SPOP mutations are present in different ethnicities, since some authors described differences according to the patients’ ancestry. We [52] and others observed different mutation frequencies between African and European patients, with SPOP mutations differing significantly between both groups. In our study, SPOP mutations were found in more than 20% of patients with African origin compared to 10% of European origin. In contrast, a recent study analyzing 720 PCa samples from six international cohorts, including Caucasian, African–American and Asian PCa patients, showed that SPOP mutations were variably frequent (4.6–14.4%), but found no association with ethnicity. Hence, the authors concluded that SPOP mutations were not associated with ethnicity, biochemical recurrence, clinical parameters or pathological parameters [53]. In light of inconsistent data, more studies are needed to make a conclusive statement.

CDK12 is a gene also implicated in DNA repair by regulation of the expression levels of different DNA repair damage response genes. CDK12 is recurrently mutated in aggressive localized PCa [52] and in mPCa [54]. CDK12 biallelic loss is associated with focal tandem duplications. Moreover, CDK12 mutations are related to increased gene fusion, which can yield neoantigens and induce strong immune infiltration, suggesting that patients with these mutations could benefit from immune checkpoint immunotherapy [54].

## 6. Androgen Receptors

PCa is a hormone-sensitive cancer, and androgen receptors (AR) play a major role in the treatment and development of PCa. For advanced PCa, ADT is the standard of care, but is decisively ineffective in castration-resistant PCa (CRPCa) [1]. Under ADT treatment, circulating testosterone indicates the androgen suppression level. In CRPCa, the activity of AR remains elevated, despite reduced androgen levels. In recent years, second-generation, AR-targeting therapies were developed in order to treat CRPCa with agents to antagonize AR and to suppress extragonadal androgen (coming from the adrenal gland, for instance) [55].

The AR gene, located on Xq11-12, is a major transcriptional regulator in the normal prostate, but also in PCa cells. The AR, a steroid hormone receptor, forms a complex with heat-shock protein 90 (HSP-90). When binding with androgen, it undergoes a change, allowing nuclear translocation, DNA binding and regulation of gene transcription [56]. Different structural variants exist, and some AR were previously implicated in aggressive tumor activity (see below). The AR gene may undergo genomic alterations such as point mutations, which can induce structural changes. These alterations, specifically seen in CRPCa, can help to understand the dependence of CRPCa on androgen-independent AR signaling.

After ligand binding, the AR is translocated into the nucleolus, forms a dimer and binds to the androgen-response element of the promoter or enhancer of target genes [57]. Furthermore, the AR dimer forms a complex with coactivator- and coregulatory proteins in different regions and regulates gene expression with diverse functions. These are located downstream of the androgen response element, including fusion genes (TMPRESS2–ERG), transcription factors (FOXP1, NKX3.1) and others. Many coactivators interacting with different AR domains are implicated in AR activation in therapy-resistant PCa. While normal AR activity on transcription is ligand-driven, AR transcript variants can encode truncated AR proteins lacking a ligand-binding domain, which can activate AR-target genes in the absence of androgens [58].

The spectrum of genes regulated by AR deserves special attention. AR has transcriptional activity and structural variants exist which play roles in the outcome of the patient. However, it is unknown to what extent individual primary PCa tumors differ in androgen sensitivity or dependence. Androgen activity is a central axis in PCa evolution, and drives most ETS fusion genes [26]. ETS fusion genes are under AR control, but the ETS fusion-positive groups have different AR transcriptional activity. More frequent events, such as androgen-regulated fusions of ERG or ETS family members, form distinct PCa groups (T2E, see above). The most frequent drivers are ERG, ETV1-4, SPOP and FOXA1 mutations [14]. TMPRSS2 is the most frequent fusion partner of all ETS fusions and is androgen-related. Tumors with SPOP or FOXA1 mutations have the highest AR transcriptional activity, as SPOP mutations deregulate AR and AR co-activators [59]. In PCa. two different changes affect the AR pathway. The output is controlled by AR mRNA and protein expression, but also by the expression and mutations of AR cofactors [60]. FOXA1, which can be mutated, is a transcription factor that targets AR and plays an obvious role in PCa oncogenesis. A subset of these mutations also harbor SPOP mutations and consequently produce high AR levels.

It must be mentioned that the AR is the most frequently aberrant gene in mCRPCa, with around 63% aberrant expression [12]. Point mutations are found in 15–30% of CRPCa, most of the mutations residing in the ligand-binding domain. These point mutations can activate AR with a specific point mutation (T878A), which also activates resistance to second generation AR agonists [61]. These mutations were detected in 13% of CRPCa patients with abiraterone resistance. A second observed mechanism was the amplification of the AR receptor, seen in up to 50% of patients with CRPCa [62]. In case of ADT, low androgen levels still exist. In case of AR amplifications, PCa cells can survive under ADT, leading to progression of CRPCa. Change in androgen biosynthesis also plays a role. For instance, during ADT, the adrenal gland still produces androgens, while CRPCa overexpresses converting enzymes, converting weak androstenedione levels into DHT to activate AR [63].

The AR splice variants (AR-Vs) have different mRNA sequences than the full-length AR (AR-FL). Around 22 AR-Vs are currently known, most of them lacking the ligand-binding domain (LBD), which is the target of existing AR therapy. Most of these AR-Vs mediate active AR signaling, which means that they can act without the presence of androgens or AR-FL [64]. One of the best-described variants is AR-V7, which also lacks the LBD. AR-V7, known to be an important factor in the treatment of CRPCa, can already be detected in primary tumors and surrounding normal prostate tissue. This is surprising, since truncated AR splice variants were proposed to be expressed predominantly in mCRPCa, and their presence was associated with hormone-therapy resistance, at least for AR-V7. However, the TCGA study showed that these splice variants are already expressed in therapy-naive primary PCa [14]. Structural alterations of AR-Vs may occur in the same allele, leading to a generation of AR-Vs displaying deletions and duplications within the AR LBD. AR-V expression can be regulated by both splicing enhancer sequences and also protein kinase pathways [65].

It could be beneficial to obtain AR-V7 data before treating patients with mCRPCa, as a recent study suggested [66]. The authors showed that patients with mCRPCa exhibiting nuclear localized AR-V7 in circulating tumor cells had better outcomes when treated by taxanes than by AR signaling inhibitors. In a follow-up study, Graf et al. [67] showed that AR-V7-positive patients who were treated with taxanes exhibited better survival, while those who were AR-V7-negative exhibited better survival when treated with AR signaling inhibitors. Therefore, in the era of emerging personalized medicine, the status of the most important splice variants could become an important clinical criterion in the future.

## 7. Conclusions

As this short overview shows, genomic changes in PCa development are extremely complex and many different factors need to be considered. One major challenge of advanced disease is that many proposed underlying mechanisms are still insufficiently understood or unclear due to contrasting results, and thus cannot yet be leveraged as refined therapeutic approaches. However, science is progressing rapidly. This review provides a snapshot of the current knowledge, but in the upcoming years, more data will be available to treat this frequent cancer more effectively.
